# Erratum: Siddagangaiah, S., Chen, C.-F., Hu, W.-C., Pieretti, N. A Complexity-Entropy Based Approach for the Detection of Fish Choruses. *Entropy* 2019, *21(10)*, 977

**DOI:** 10.3390/e21121140

**Published:** 2019-11-22

**Authors:** Shashidhar Siddagangaiah, Chi-Fang Chen, Wei-Chun Hu, Nadia Pieretti

**Affiliations:** 1 Underwater Acoustic Laboratory, Department of Engineering Sciences and Ocean Engineering, National Taiwan University, Taipei 106, Taiwan; william_hu@outlook.com; 2 Department of Life and Environmental Sciences, Polytechnic University of Marche, 60131 Ancona, Italy; nadia.pieretti@gmail.com

We have found an error in the order of one author’s name and surname in the article recently published in *Entropy* [1]:

Shashidhar Siddagangaiah ^1,^*, Chi-Fang Chen ^1,^*, Wei-Chun Hu ^1^ and Pieretti Nadia ^2^

that should be corrected to the following order in this paper [1]:

Shashidhar Siddagangaiah ^1,^*, Chi-Fang Chen ^1,^*, Wei-Chun Hu ^1^ and Nadia Pieretti ^2^

The authors would like to apologize for any inconvenience caused to the readers by these changes.
