# Supplementary material for: A Complexity-Entropy Based Approach for the Detection of Fish Choruses
Source: Entropy (Basel). 2019 Oct 6;21(10):977. doi: 10.3390/e21100977 (PMC7514308; doi:10.3390/e21100977)
Supplement: Supplementary file 1 [file entropy-21-00977-s001.docx]

Compute C-H

Acoustic Data

Use ‘statcomp’ R package to compute C and H using the command *global-complexity*(X=’xxx.wav’, *d* = 3,…,7)

Use ‘tuneR’ package in R to read the hourly acoustic recordings in .wav format

**Figure S1**: Flowchart for evaluation of C and H for hourly passive acoustic monitored data.


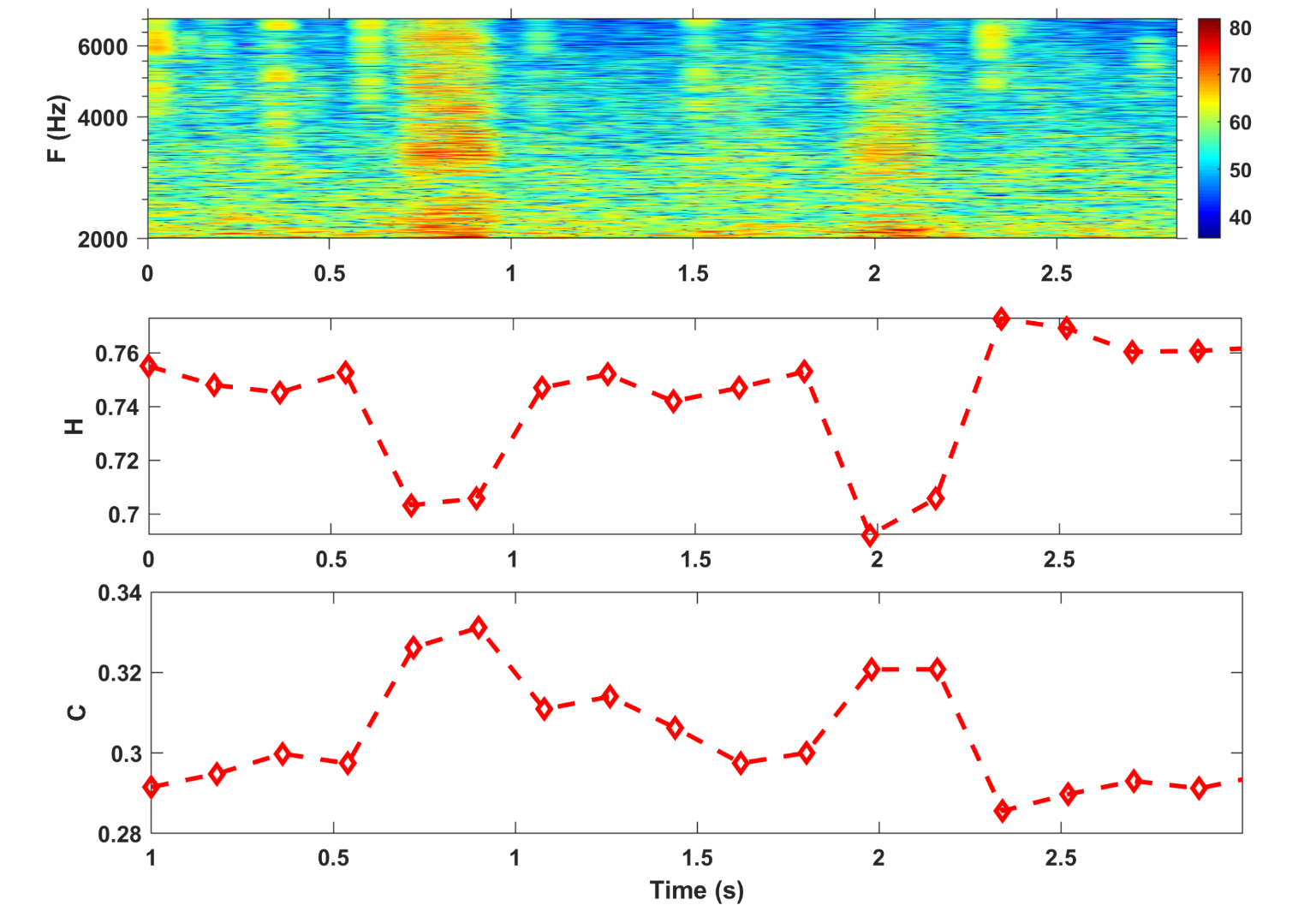


**(c)**

**(b)**

**(a)**

**Figure S2**. Spectrogram showing fish calls at ~0.7s and ~2s. The H and C responds to the fish calls by dipping and peaking, respectively. C and H are computed over the time steps of 0.18s.


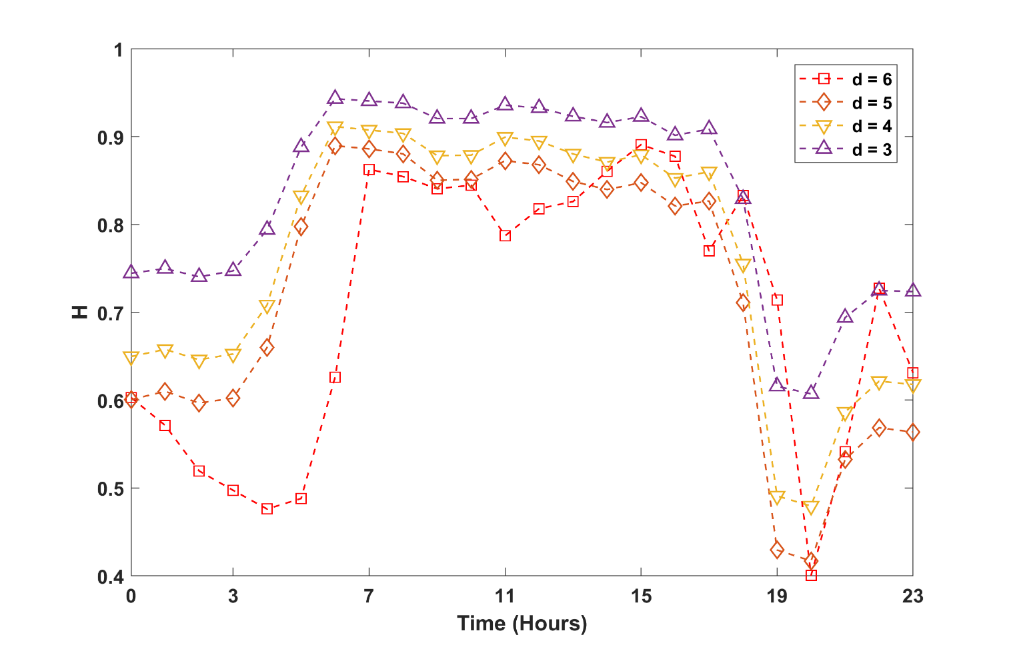

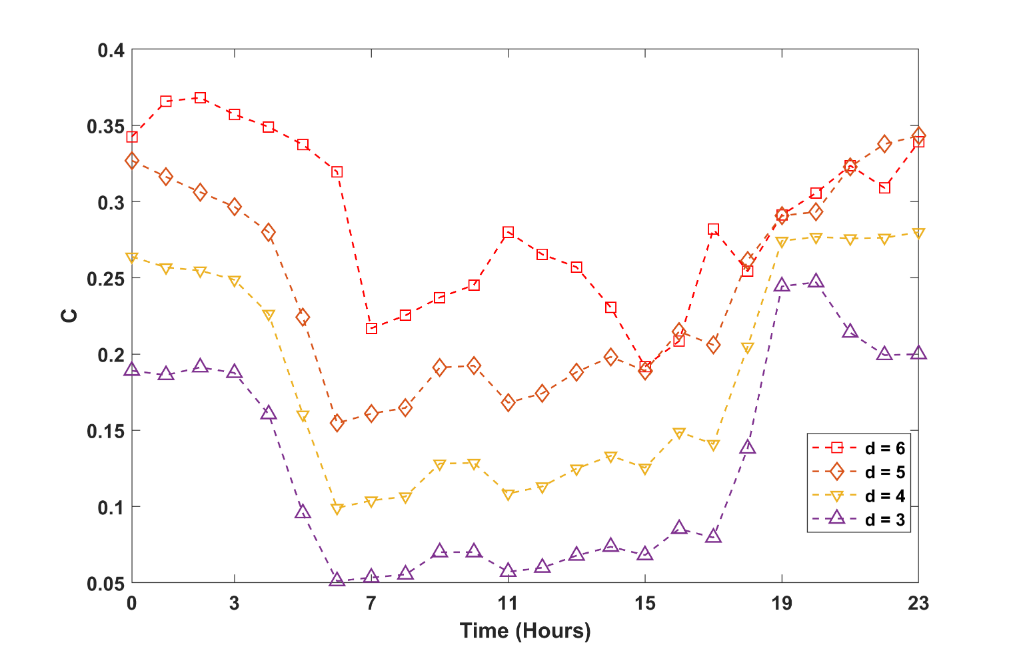


**(b)**

**(a)**

**Figure S3: (a, b)** Hourly variation of H and C for the varying d = 3,..,6 computed for 24-hrs acoustic recording. We verified the robustness of the choice of the embedding dimension d ∈ {3, 4, 5, 6} by applying it to the 24-hr recordings on 26 April 2016 (Figure 4a). The H and C for varying d have troughs and peaks respectively occurring in correspondence of fish choruses at dawn and dusk. However, it can be observed that at a higher d corresponds a lower H (Entropy) and a higher C (Complexity).


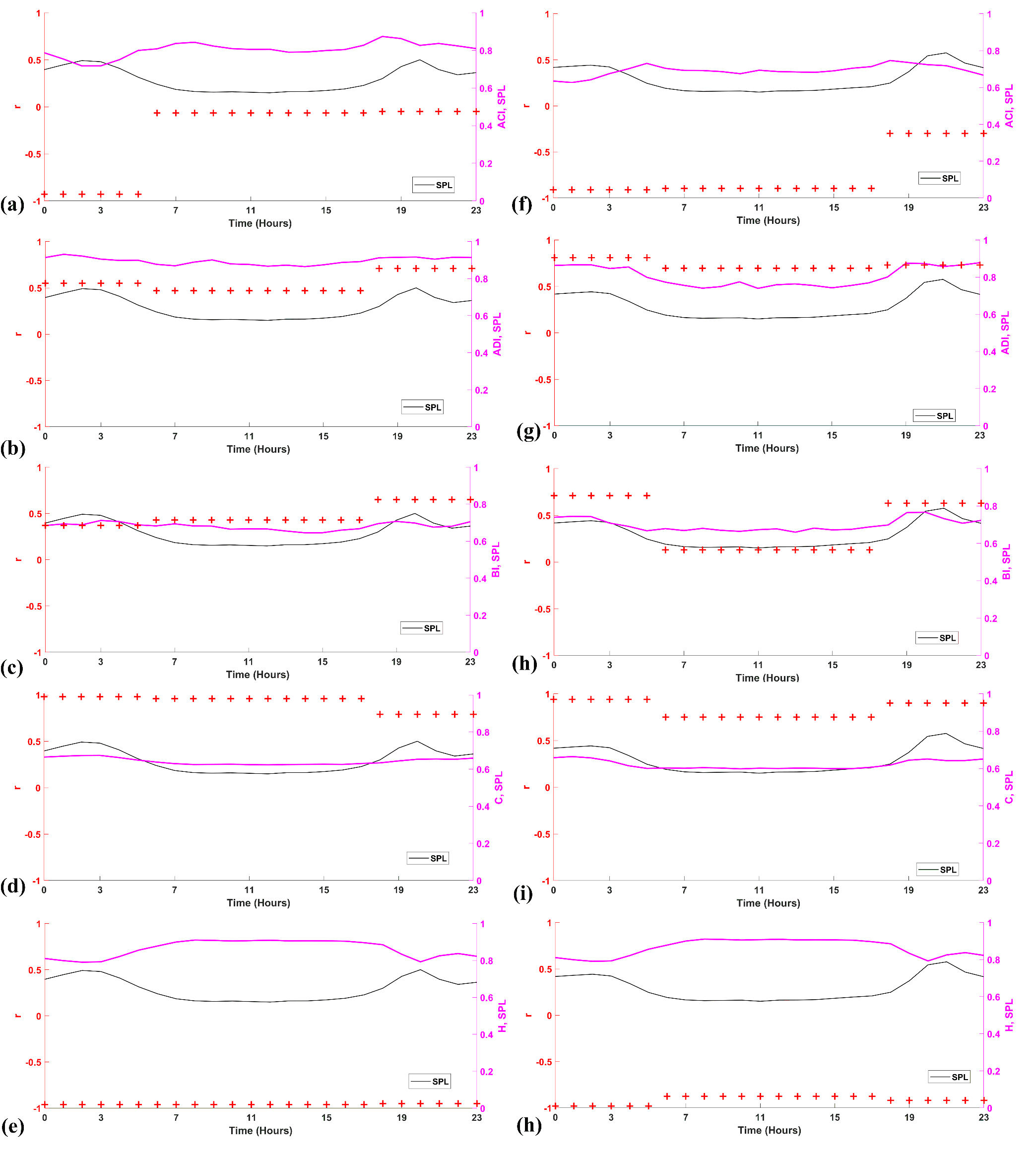


**Figure S4.** Graphs show the normalized hourly means of the indices at A1 (in pink) and the corresponding hourly correlation coefficient (r) (in red). As a comparison, normalized hourly mean SPL_500 – 2500 Hz_ are also shown (in black).


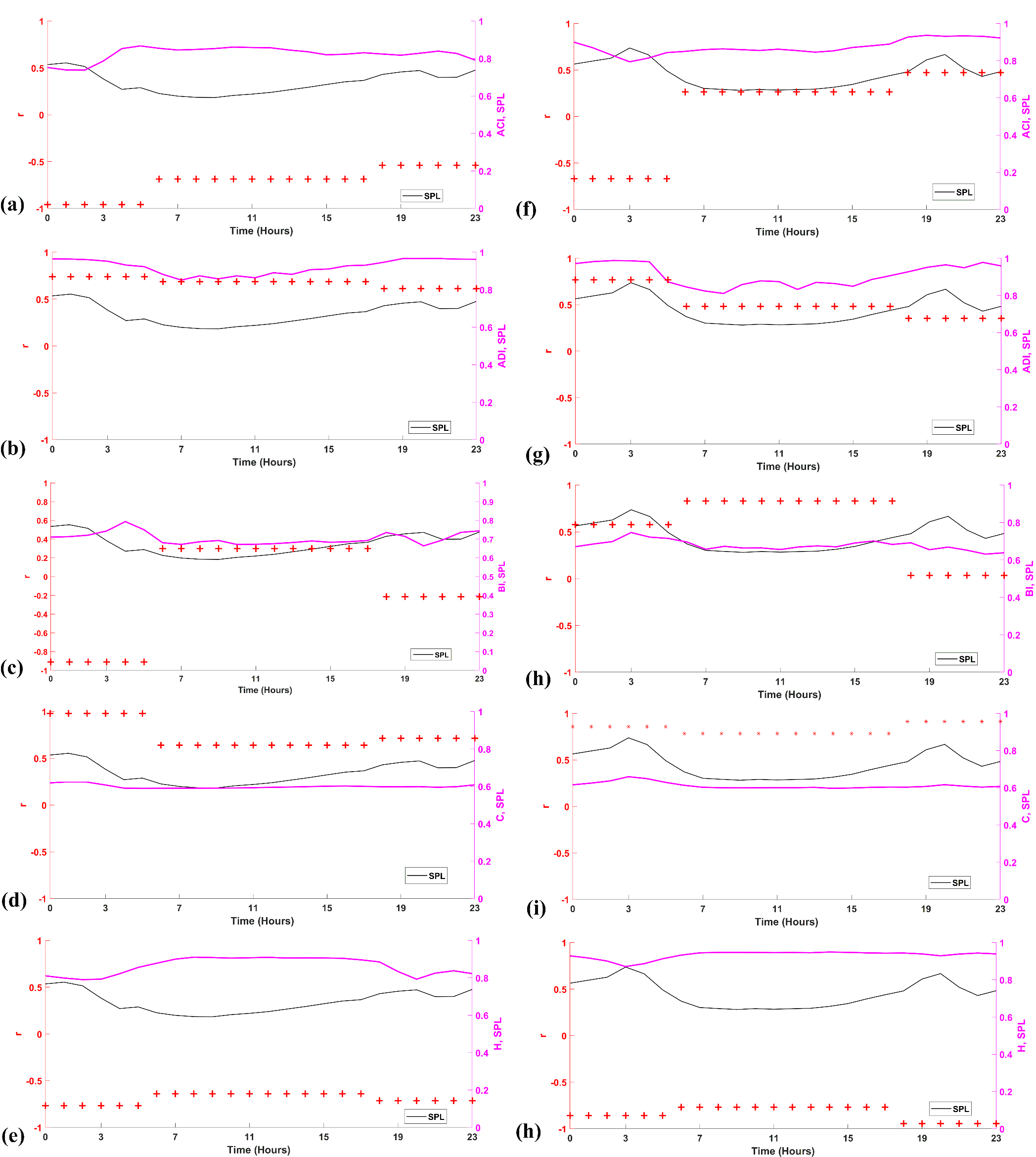


**Figure S5.** Graphs show the normalized hourly means of the indices at N1 (in pink) and the corresponding hourly correlation coefficient (r) (in red). As a comparison, normalized hourly mean SPL_500 – 2500 Hz_ are also shown (in black).


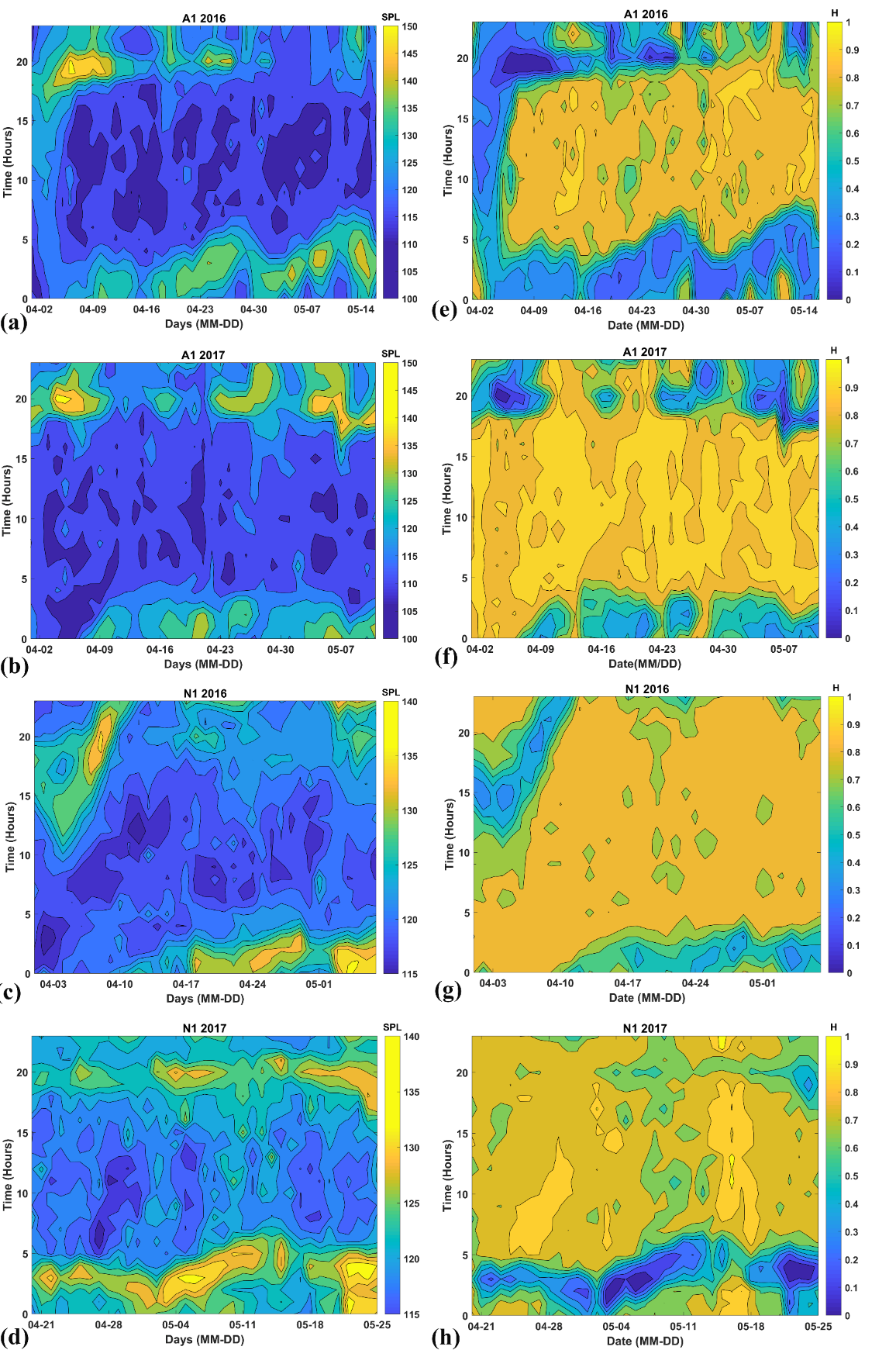


**Figure S6**: Fish chorusing trend on 3-D contour plot during spring 2016 and 2017 based on SPL_500-2500Hz_ (**a-d**) and the corresponding normalized entropy (H), which is anticorrelated with the fish chorusing (**e-h**).

**S7** - R code for computation of H and C for hourly passive acoustic monitored data

library("tuneR")

library("statcomp")

fnam=file.path("F:/Folder");

filist=list.files(fnam, recursive=TRUE, pattern="wav");

filist1=paste(fnam, "/", filist, sep="");

nfiles=length(filist1);

x1<-0;

A2<-0;

A1<-0;

vec1<-0;

for (i in 1:nfiles){

inname=filist1[i]

ywave=readWave(inname);

vec1 <- ywave@left;

t <- global_complexity(x = vec1, ndemb = 6)

A1[i] = t[1]; %% Entropy

A2[i] = t[2]; %% Complexity

print(i)

}
